# Pre- and Post-Operative Cone Beam Computed Tomography Assessment of the Temporomandibular Joint in Patients with Orthognathic Surgery

**DOI:** 10.3390/diagnostics14131389

**Published:** 2024-06-29

**Authors:** Thomas J. Vogl, Wael Zyada, Rania Helal, Nagy N. Naguib, Neelam Lingwal, Nour-Eldin A. Nour-Eldin

**Affiliations:** 1 Department of Diagnostic and Interventional Radiology, University Hospital Frankfurt, Goethe University Frankfurt, Theodor-Stern-Kai 7, 60590 Frankfurt, Germany; raniahelal@med.asu.edu.eg (R.H.); nour410@hotmail.com (N.-E.A.N.-E.); 2 Department of Diagnostic and Interventional Radiology, Ain Shams University Hospital, Ain Shams University, Cairo 11566, Egypt; 3 Department of Diagnostic and Interventional Radiology, Bad Salzungen Hospital, 36433 Bad Salzungen, Germany; nagynnn@yahoo.com; 4 Department of Diagnostic and Interventional Radiology, Alexandria University Hospital, Alexandria University, Alexandria 21526, Egypt; 5 Department of Biostatistics and Mathematical Modelling, University Hospital Frankfurt, 60590 Frankfurt, Germany; lingwal@med.uni-frankfurt.de; 6 Department of Diagnostic and Interventional Radiology, Cairo University Hospital, Cairo University, Cairo 11956, Egypt

**Keywords:** orthognathic surgical procedures, dentofacial deformity, temporomandibular joint, cone beam computed tomography

## Abstract

This study aimed to compare the pre- and post-operative temporomandibular joint (TMJ) condylar position in dentofacial deformity (DFD) patients who had orthognathic surgeries using cone beam computed tomography (CBCT). A retrospective study evaluating the pre- and post-operative CBCT for 79 DFD patients (equivalent to 158 TMJs) (mean age = 26.62 ± 9.5 years) with a bilateral sagittal split osteotomy with or without Le Fort I surgeries (*n* = 29 Class II DFD, *n* = 50 Class III DFD) was performed. This included the compartmental measurement of TMJ spaces, in addition to the measurement of intercondylar distances and angles. Condylar position centricity was assessed using the Pullinger and Hollender formula. Clinical data were analysed for DFD class, the type of surgery and post-operative CBCT timing. Pre- and post-operative measurements were compared statistically using a paired *t*-test, Wilcoxon signed-rank test, and Stuart–Maxwell test. TMJ condyles tended to relocate post-operatively in a posterosuperior position with internal rotation in Class II DFD and a superior position with internal rotation in Class III DFD. However, the overall changes were within <0.5 mm translation and <4° rotation and the number of concentrically positioned condyles (according to the Pullinger and Hollender formula) did not change significantly. Orthognathic surgery is associated with minor post-operative translational and rotational condylar positional changes in Class II and III DFDs.

## 1. Introduction

Dentofacial deformities (DFDs) are significant changes in the normal proportions of the maxillomandibular complex that affect facial profile, occlusion, and the integrity of the temporomandibular joint (TMJ). They have several forms; the most common are sagittal plane deformities such as prognathism and retrognathism, which are the forward (prognathic) or backward (retrognathic) displacement of one or both jaws relative to the base of the skull [1]. Temporomandibular joint morphology may be compromised in patients with dentofacial abnormalities due to abnormal forces exerted on the joint. These changes may predispose to various temporomandibular joint pathologies such as disc perforation, disc displacement, and osteoarthritis [2].

In the last few decades, orthognathic surgery has become the standard treatment for dentofacial deformities. It is generally considered a safe treatment method with a low rate of complications. The most frequent method for orthognathic operations is a bilateral sagittal split osteotomy (BSSO) with and without a Le Fort I osteotomy [3]. This means the positional relationship of the lower jaw and/or upper jaw is changed by means of a conversion osteotomy [4].

The radiological assessment of the TMJ can be done using panoramic radiography, conventional radiography, computed tomography (CT) and magnetic resonance imaging (MRI). Cone beam CT (CBCT) is now considered a valuable tool for imaging in the context of orthognathic operations [5,6]. With a relatively low radiation dose, CBCT can replace X-rays and CT in TMJ imaging [7,8]. In addition, CBCT can enable a 3-D cephalogram to accurately display important landmarks and aid in virtual surgical preoperative planning. In post-operative imaging, CBCT also offers a high-resolution visualization of high-contrast structures with low susceptibility to metallic artifacts [9,10].

To date, some studies have examined changes in condylar position after orthognathic surgery; however, it is still unclear how orthognathic surgical procedures change condylar position and whether these changes deviated significantly from preoperative temporomandibular joint position [11,12,13,14].

The purpose of this study was to retrospectively examine and compare temporomandibular condylar position and joint spaces in the pre-operative and post-operative CBCT of patients with Class II and Class III DFDs who underwent BSSO ± Le Fort I surgery to investigate whether changes in mandibular position in orthognathic surgeries cause significant changes in TMJs or not.

## 2. Materials and Methods

### 2.1. Study Design and Population

**Figure 1:**
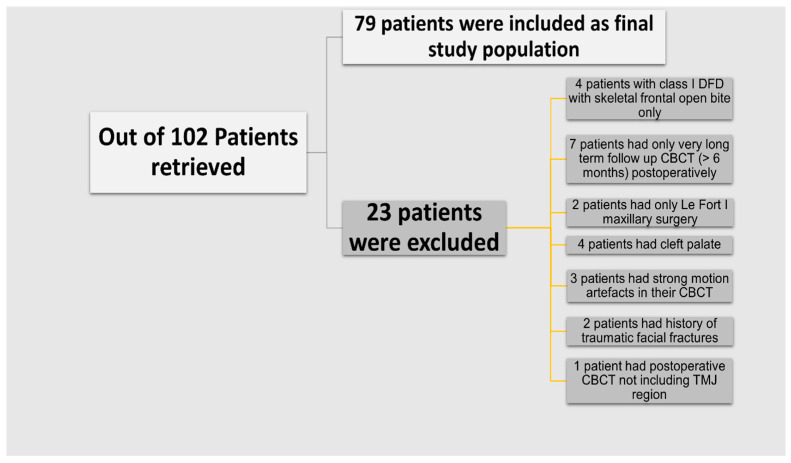
Flowchart showing included study population and exclusion criteria.

This single-centre retrospective study was approved by the local institutional review board with a waiver for informed consent. Patients who had BSSO ± Le Fort surgeries for the correction of DFDs in addition to CBCT examinations before and after surgery (with a median of 6 weeks post-operatively) between January 2017 and October 2021 were included regardless of their age or gender. Out of 102 patients retrieved, 23 patients were excluded as shown in Figure 1. The final study population consisted of 79 patients with a mean age of 26.62 ± 9.5, including, 29 (36.7%) patients with Class II DFD (mandibular retrognathism/maxillary prognathism or both), and 50 (63.3%) patients with Class III DFD (mandibular prognathism/maxillary retrognathism or both).

### 2.2. Operative and Post-Operative Data Evaluation

Operative and post-operative records of the patients were gained using the local institutional database (ORBIS, Hospital information system (HIS), Agfa Health Care Systems Group, Bonn, Germany). Analysed patients’ data included age, gender, the class of DFD, the type and date of orthognathic surgery, the date of pre- and post-operative CBCT studies, the presence of cleft palate and previous history of facial fractures.

### 2.3. Cone-Beam CT (CBCT)

CBCT scans were obtained from bilateral TMJs in a closed mouth intercuspation position with 120 KV and 7.1 mA in a field of view of 230 mm/160 mm using a PLANMECA ProMax-3D Max CBCT device (Planmeca Oy, Helsinki, Finland).

### 2.4. Image Evaluation

Image data sets were evaluated after anonymization by an experienced head and neck radiologist (10 years of experience) and an experienced oral surgeon (11 years of experience) in consensus, being blinded to the clinical data (age and gender) of the patients. The evaluation was carried out on certified diagnostic screens (RadiForce RX240; Eizo, Ishikawa, Japan) with a special dedicated PACS viewer (Centricity 7.0; GE Healthcare, Germany). Measurements were taken on the three main reformatted projections (axial, sagittal and coronal). The pattern of measurement was adopted from Chen et al. and Draenert et al. [14,15].

#### 2.4.1. Axial Projection: Measurements Were Conducted on the Slice with the Largest Condylar Dimensions in the Axial Plane (Figure 2)

- Intercondylar distance: defined as the distance between midpoint of right and left condylar heads.
- Intercondylar angle: defined as the angle formed between lines connecting medial and lateral poles of the condylar heads on both sides.

**Figure 2:**
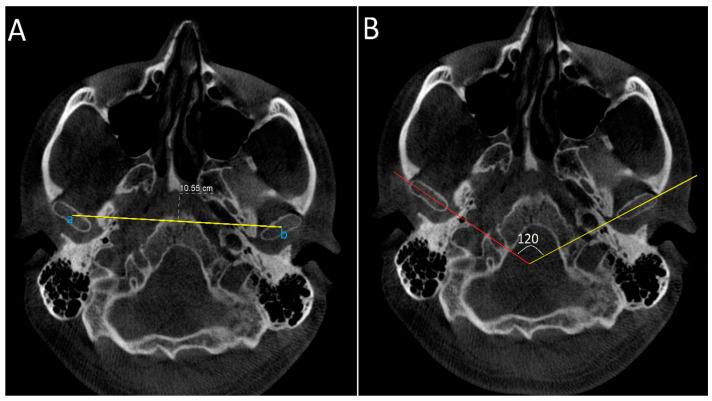
Axial CBCT images of a patient showing the measurement of the axial intercondylar distance and angle. In image (**A**), the axial intercondylar distance equals the yellow line joining point (a), equivalent to the centre of the right condyle, and point (b), equivalent to centre of the left condyle. In Image (**B**), the axial intercondylar angle is the angle between the lines joining the medial and lateral pole of the right condyle (red line) and the line joining the medial and lateral poles of the left condyle (yellow line).

#### 2.4.2. Oblique Sagittal Projection (Figure 3): Measurements Were Carried out on the Image in the Sagittal Plane Corresponding to the Middle Point of the Condyle in the Axial Plane and Coronal Plane on the Slices with the Largest Condylar Dimensions

- The uppermost point of the mandibular fossa is set as point (a) from which two tangent lines are drawn to join the most anterior and most posterior margins of the condylar head. By drawing perpendicular lines to these tangents, anterior (AJS) and posterior (PJS) joint spaces were measured. Another line joining point (a) and the most superior part of the condyle was used to measure the superior joint space (SJS).

- Condylar position was assessed after that using the formula according to the method of Pullinger and Hollender [16]:
  Condylar position ratio = ln (PJS/AJS)
  Ratio = ±0.25, condyle in concentric position.
  Ratio < −0.25, condyle in posterior position.
  Ratio > 0.25, condyle in anterior position.

**Figure 3:**
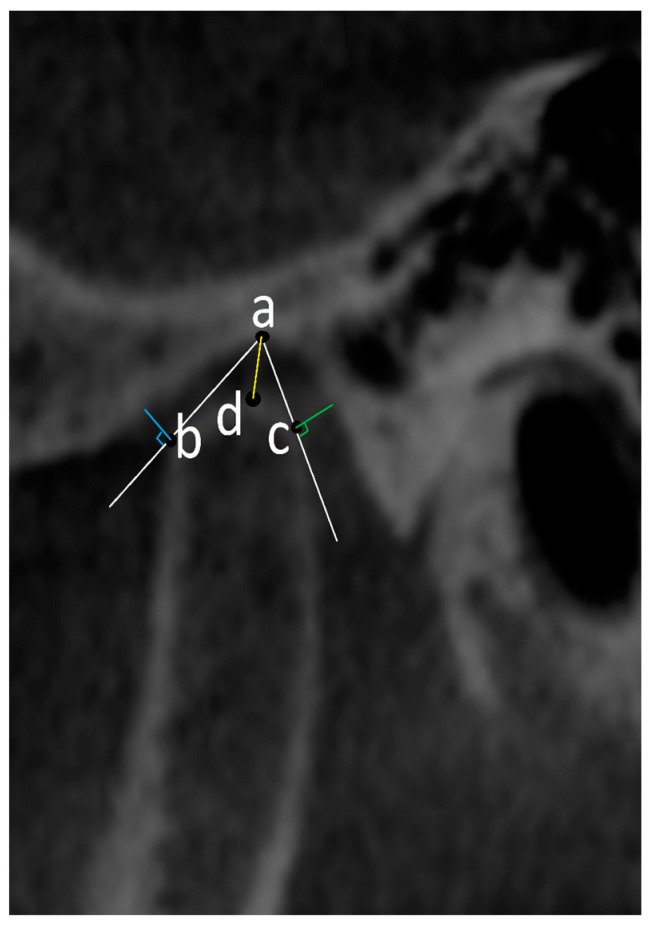
Oblique sagittal reformatted CBCT image showing the measurement of the anterior joint space (AJS), posterior joint space (PJS), and superior joint space (SJS). The measurements were carried out on the image in the sagittal plane corresponding to the middle point of the condyle on the axial plane and coronal planes on the slices with the largest condylar dimensions. Point (a) is drawn at the uppermost point of the mandibular fossa from which two tangents (white lines) are drawn on the anterior (b) and posterior (c) margins of the condylar head and using a line perpendicular to these tangents, the AJS (blue line) and PJS (green line) are measured. Another line joining point (a) and the most superior part of the condyle (d) was used to measure the SJS (yellow line).

#### 2.4.3. Coronal Projection (Figure 4 and Figure 5): Measurements Were Carried out on the Image in the Coronal Plane Corresponding to the Middle Point of the Condyle on the Axial and Sagittal Planes on the Slices with the Largest Condylar Dimensions

- Medial joint space (MJS): distance between the medial pole of the mandibular condyle and medial wall of glenoid fossa.
- Lateral joint space (LJS): distance between the lateral pole of the mandibular condyle and lateral wall of glenoid fossa.
- Intercondylar distance: defined as distance between the midpoint of the right and left condylar heads.
- Intercondylar angle: defined as the angle formed between lines connecting the medial and lateral poles of the condylar heads on both sides.

**Figure 4:**
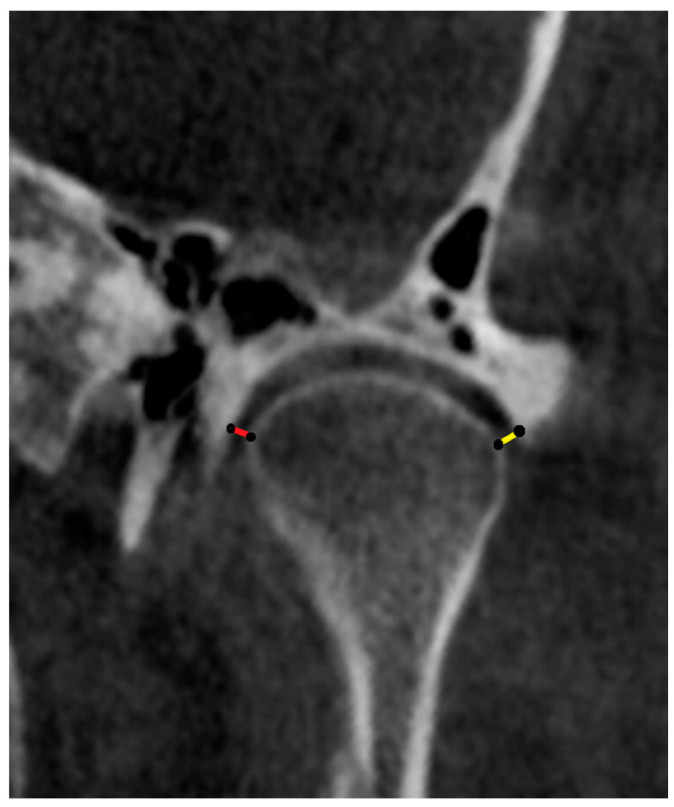
Coronal reformatted CBCT image of a patient showing measurement of the medial (MJS) and lateral (LJS) joint spaces. The measurements were carried out on the image in the coronal plane corresponding to the middle point of the condyle on the axial and sagittal plane on the slices with the largest condylar dimensions. MJS corresponds to the distance between the medial pole of the mandibular condyle and the medial wall of the glenoid fossa (red line). LJS corresponds to the distance between the lateral pole of the mandibular condyle and the lateral wall of the glenoid fossa (yellow line).

**Figure 5:**
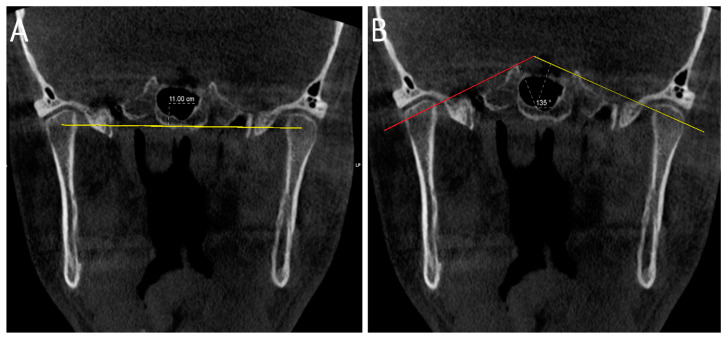
Coronal reformatted CBCT images of a patient showing measurement of the intercondylar angle and distance. In image (**A**), the coronal intercondylar distance equals the yellow line joining the centre of the right and left condyles, In Image (**B**), the coronal intercondylar angle is the angle between the lines joining the medial and lateral pole of the right condyle (red line) and the line joining the medial and lateral poles of the left condyle (yellow line).

### 2.5. Statistical Analysis

Statistical analysis was executed using IBM SPSS Statistics software (version 26.0) for Windows (IBM Corp., Armonk, NY, USA). Qualitative data were presented as numbers, and percentages. Quantitative data were shown as a median with inter-quartile range (IQR) when not normally distributed and a mean ± standard deviation (SD) when normally distributed (after being tested with Kolmogorov–Smirnov test for normality). For age, the mean ± SD and range were also described.

Paired *t*-Test (normally distributed) and Wilcoxon paired signed-rank test (non-normally distributed) were used to compare paired pre- and post-treatment quantitative measurements. The marginal homogeneity/Stuart–Maxwell test was used to compare nominal data groups in paired data between pre- and post-treatment. All tests were two-tailed and *p* ≤ 0.05 was considered significant.

## 3. Results

The included study population was 79 patients (equivalent to 158 TMJs). The mean age was 26.62 ± 9.5 years. The different patient-related characteristics are shown in (Table 1).

### Radiological Assessment of TMJ Joint Spaces and Condylar Position Analysis

We measured TMJ joint spaces in the mid sagittal plane (AJS, PJS, and SJS) and in the coronal plane (LJS and MJS) for the entire study population. This revealed statistically significant differences between pre- and post-operative measurements in all measured spaces on both sides except for the AJS (Table 2). In the group with Class II DFD, all joint spaces’ measurements were statistically significant when comparing pre- and post-operative measurements except for the AJSs (Table 3). However, in the group with Class III DFD, in addition to the bilateral AJSs, changes in PJS bilaterally were also statistically insignificant (Table 3). 

Using Pullinger and Hollender’s formula, the assessment of the centricity of the condyle in the condylar fossa also revealed overall statistically significant changes between pre- and post-operative measurements in the entire study population, and in Class II DFD, while changes between pre- and post-operative measurements in Class III DFD groups were statistically insignificant. Despite significant change in the overall ratio, the number of concentric, anteriorly positioned and posteriorly positioned condyles did not show significant change (Table 4).

The pre- and post-operative comparison of intercondylar distance in both axial and coronal planes and intercondylar angle in the coronal plane in the entire study population, Class II, and Class III were statistically insignificant. However, the intercondylar angle significantly decreased in all groups in post-operative measurements compared to the pre-operative ones (Table 5).

## 4. Discussion

One of the major and common concerns in patients with DFDs is the associated TMJ dysfunction. The repositioning of bone segments during orthognathic surgeries may lead to mandibular condyle positional changes that can affect TMJ [17]. In the current study, we encountered a statistically significant decrease in the MJSs, LJSs, and SJSs in the post-operative studies of patients with DFDs Class II and III separately. These joint space changes indicated that the condyle has moved superiorly in the mandibular fossa, associated with a decrease in both medial and lateral joint spaces. Furthermore, PJSs decreased significantly in the Class II DFD group; however, this was not statistically significant in Class III DFD. We also calculated a significant decrease in the intercondylar angle in the axial plane without a significant change in the intercondylar distance or the coronal intercondylar angle; this indicated an associated inward rotation of the condyles. This inward rotation is concomitant with the results of An et al. in Class III [18] and Goncalves et al. in Class II [11]. These results are also concordant with results of Chen et al. [15] that showed overall posterosuperior movement in comparison with the pre-operative position in Class II patients at 3 months post-operatively and this position remained stable after 1-year follow-up. Similarly, the results of Harris et al. [12] showed that most condyles tended to displace medially, posteriorly and superiorly, and angle medially, 8 weeks after BSSO advancement for Class II DFD. On the contrary, Han et al. [19] stated that condyles in Class III patients were displaced laterally with inward and inferior rotation immediately post-operatively, which was followed by relative medial and superior displacement in the 3–6 month follow-up period, which supports the early follow-up results of Chen et al. and Ma et al. [15,20]. Few studies have stated that there were no positional changes between pre- and post-operative condylar positions [14].

The difference between studies’ results can be attributed to different time intervals between measurements [17]. The inferior displacement can be expected in the immediate post-operative stage because of the postsurgical soft tissue oedema, or condylar sagging associated with the application of muscle relaxants under general anaesthesia. After the resolution of oedematous changes and removal of the surgical stent, condyles tend to move under the force of masticatory muscles and the strain of temporomandibular ligament. Also, since tissues are extended in surgery, especially in mandibular advancement, the resulting post-operative contraction may lead to the posterior displacement of the condyle [17]. The posterior displacement could be also explained in some cases by surgical manual manipulation over proximal segments during the surgical procedure [13]. In the current study, the assessment was done with a median duration of only 6 weeks post-operative; however, this duration was enough for the resolution of the oedematous post-operative changes and the results are concomitant with the studies with longer term follow-up [15,19]. 

Park et al. stated that the presence of the condylar head in the centre of the articular fossa may prevent post-operative structural changes in TMJ [21]. According to the formula of Pullinger and Hollender [16], although there was a relative shift of condyles posteriorly in Class II, the overall change in the percentage of the centric positioned condyles did not change significantly in Class II nor Class III patients. Kim et al. reported a shift of the condyles posteriorly in the post-operative CBCT in Class III patients that tended to return to their original position in follow-up and did not affect the joint stability [22]. Also, the reported changes in all joint spaces and intercondylar angles in the current study were less than 0.5 mm and 4°, respectively, which were concomitant with the results of Chen et al. and Han et al. [15,19]. This may show that all changes in condylar position as a result of orthognathic surgery were minimal and may play a minor role in post-operative TMJ dysfunction.

Nevertheless, there were some limitations associated with this study that might compromise the general applicability of the study results. The limited sample size and the relatively short-term follow-up, however, can be attributed to the small number of patients with DFDs. Thus, larger studies with short- and long-term follow-up can still be advised to confirm our results. In post-operative CBCT studies, the presence of osteotomies and metallic prosthesis made it difficult to totally blind the examiners to avoid bias. Also, the internal derangements of the TMJ could not be well assessed using CBCT alone, as the articular disc and the articular soft tissue are not visualized. Future research including both CBCT and MRI could be also recommended.

This study extended our knowledge regarding the impact of orthognathic surgeries on TMJ condylar position. Maxillofacial surgeons are encouraged to discuss with their patients that major change in TMJ condylar position is not an expected outcome of orthognathic surgery in Class II and Class III dentofacial deformities. Future studies are encouraged to compare TMJ clinical conditions with assessment using MRI and CBCT before and after surgery.

## 5. Conclusions

In conclusion, data suggest that condyles tend to relocate in a posterosuperior position with internal rotation and superior position with internal rotation in Class II and Class III DFDs, respectively, after BSSO (±Le Fort I) orthognathic surgeries in comparison to pre-operative position. However, the overall changes were within <0.5 mm translation and <4° rotation. 

## Figures and Tables

**Table 1:** Patients’ baseline characteristics.

		Class II DFD *n* = 29 (36.7%)	Class III DFD *n* = 50 (63.3%)	Total *n* = 79
Age (Years)	Mean ± SD	28.3 ± 8.2	25.7 ± 10.2	26.62 ± 9.5
	Range	16–52	16–58	16–58
	Median (IQR)	28 (10)	20.5 (12)	24 (13)
Gender	Male	13 (44.8%)	25 (50%)	38 (48.1%)
	Female	16 (55.2%)	25 (50%)	41 (51.9%)
Surgical Approach	BSSO	8 (27.6%)	11 (22%)	19 (24.05%)
	BBSO + Le Fort I	21 (72.4%)	39 (78%)	60 (75.95%)

DFD, dentofacial deformity; SD, standard deviation; IQR, interquartile range; *n*, number; BBSO, bilateral sagittal split osteotomy.

**Table 2:** Radiological assessment analysis of temporomandibular joint spaces in the study population (in mm).

			Pre-Operative	Post-Operative	*p* Value
Oblique Sagittal View	AJS (right)	Median (IQR)	2.1 (1.1)	2.2 (0.9)	0.13 ^‡^
	AJS (left)	Median (IQR)	2.1 (1.2)	2.1 (1)	0.26 ^‡^
	SJS (right)	Median (IQR)	2.5 (1.5)	2.3 (1.1)	**0.005 ^‡^**
	SJS (left)	Mean ± SD	2.8 ± 1.1	2.6 ± 1.2	0.008 *
	PJS (right)	Median (IQR)	2.2 (1.4)	2 (1)	**0.01 ^‡^**
	PJS (left)	Median (IQR)	2.3 (1.2)	1.9 (1.3)	**0.002 ^‡^**
Coronal View	LJS (right)	Median (IQR)	2.1 (1.2)	1.9 (1.2)	**0.02 ^‡^**
	LJS (left)	Mean ± SD	2.3 ± 1.01	2.04 ± 0.95	0.006 *
	MJS (right)	Median (IQR)	2.1 (1)	1.9 (1.5)	**0.001 ^‡^**
	MJS (left)	Mean ± SD	2.1 ± 0.6	1.9 ± 0.85	0.008 *

AJS, anterior joint space; SJS, superior joint space; PJS, posterior joint space; LJS, lateral joint space; MJS, medial joint space; *p* values are written in bold when statistically significant; SD, standard deviation; IQR, interquartile range; * Paired *t*-test; ^‡^ Wilcoxon signed rank test; *p* values are written in bold when statistically significant.

**Table 3:** Radiological assessment analysis of temporomandibular joint spaces in Class II and III dentofacial deformity.

				Pre-Operative	Post-Operative	*p* Value
Class II	Oblique Sagittal View	AJS (right)	Mean ± SD	2.3 ± 1.1	2.4 ± 1	0.36 *
		AJS (left)		2.3 ± 1	2.3 ± 1.1	0.4 *
		SJS (right)		2.7 ± 1	2.5 ± 1	0.01 *
		SJS (left)		2.9 ± 1.1	2.7 ± 1.1	0.01 *
		PJS (right)		2.4 ± 1	2.1 ± 0.95	0.02 *
		PJS (left)	Median (IQR)	2.3 (1.2)	2 (1.2)	**0.003 ^‡^**
	Coronal View	LJS (right)	Mean ± SD	2.3 ± 1	2.1 ± 1.1	0.04 *
		LJS (left)		2.3 ± 1	2.1 ± 0.9	0.008 *
		MJS (right)		2.3 ± 0.8	2 ± 0.9	0.003 *
		MJS (left)		2.2 ± 0.68	1.9 ± 0.8	0.002 *
Class III	Oblique Sagittal View	AJS (right)	Median (IQR)	1.9 (1)	2 (0.7)	0.58 ^‡^
		AJS (left)		2 (1.2)	1.9 (1.1)	0.31 ^‡^
		SJS (right)		2.4 (1.1)	2.2 (1)	**0.004 ^‡^**
		SJS (left)	Mean ± SD	2.6 ± 1.1	2.4 ± 1.1	0.02 *
		PJS (right)		2.1 ± 0.81	1.98 ± 0.82	0.3 *
		PJS (left)	Median (IQR)	2 (1)	1.9 (1.2)	0.296 ^‡^
	Coronal View	LJS (right)	Median (IQR)	1.9 (1.1)	1.7 (0.7)	**0.04 ^‡^**
		LJS (left)		1.95 (1.3)	1.8 (1.2)	**0.02 ^‡^**
		MJS (right)		1.9 (0.7)	1.7 (0.9)	**0.005 ^‡^**
		MJS (left)	Mean ± SD	1.99 ± 0.59	1.76 ± 0.66	<0.001 *

AJS, anterior joint space; SJS, superior joint space; PJS, posterior joint space; LJS, lateral joint space; MJS, medial joint space; *p* values are written in bold when statistically significant; SD, standard deviation; IQR, interquartile range; * paired *t*-test; ^‡^ Wilcoxon signed-rank test; *p* values are written in bold when statistically significant.

**Table 4:** Position of condyle according to the Pullinger and Hollender Formula (in (PJS/AJS)).

			Right Side		Left Side				
			Pre-Operative	Post-Operative	*p* Value		Pre-Operative	Post-Operative	*p* Value
Study population		Median (IQR)	0.078 (0.79)	−0.0445 (0.69)	**0.01 ^‡^**	Median (IQR)	0.07 (0.64)	0 (0.71)	**0.004 ^‡^**
	Condylar position	Concentric	25 (31.6%)	30 (38.0%)	0.82 ^§^	Concentric	33 (41.8%)	31 (39.2%)	0.22 ^§^
		Anterior	29 (36.7%)	21 (26.6%)		Anterior	26 (32.9%)	19 (24.1%)	
		Posterior	25 (31.6%)	28 (35.4%)		Posterior	20 (25.3%)	29 (36.7%)	
Class II DFD		Median (IQR)	0.13 (0.81)	−0.19 (0.78)	**0.002 ^‡^**	Mean ± SD	0.18 ± 0.5	−0.24 ± 0.52	<0.001 *
	Condylar position	Concentric	9 (31%)	8 (27.6%)	0.26 ^§^	Concentric	8 (27.6%)	9 (31%)	0.32 ^§^
		Anterior	12 (41.4%)	8 (27.6%)		Anterior	14 (48.3%)	6 (20.7%)	
		Posterior	8 (27.6%)	13 (44.8%)		Posterior	7 (24.1%)	14 (48.3%)	
Class III DFD		Mean ± SD	−0.028 ± 0.56	−0.061 ± 0.54	0.63 *	Median (IQR)	0.04 (0.53)	0.036 (0.57)	0.66 ^‡^
	Condylar position	Concentric	16 (32%)	22 (44%)	0.27 ^§^	Concentric	25 (50%)	22 (44%)	0.46 ^§^
		Anterior	17 (34%)	13 (26%)		Anterior	12 (24%)	13 (26%)	
		Posterior	17 (34%)	15 (30%)		Posterior	13 (26%)	15 (30%)	

*p* values are written in bold when statistically significant; SD, standard deviation; IQR, interquartile range; * paired *t*-test; **^‡^** Wilcoxon signed-rank test; ^§^ marginal homogeneity test/Stuart–Maxwell test; DFD, dentofacial deformity; *p* values are written in bold when statistically significant.

**Table 5:** Radiological assessment analysis of intercondylar distance (in cm) and intercondylar angle.

				Pre-Operative	Post-Operative	*p* Value
Whole Study population	Axial	Intercondylar angle	Mean ± SD	139.5 ± 19.6	136.6 ± 19.9	0.001 *
		Intercondylar distance	Median (IQR)	10.03 (0.83)	9.997 (0.83)	0.62 ^‡^
	Coronal	Intercondylar angle		150 (19)	151 (23)	0.71 ^‡^
		Intercondylar distance		9.86 (0.67)	9.84 (0.76)	0.08 ^‡^
Class II DFD	Axial	Intercondylar angle	Mean ± SD	139.61 ± 19.27	136.55 ± 19.53	<0.001 *
		Intercondylar distance		10.06 ± 0.65	10.02 ± 0.61	0.66 *
	Coronal	Intercondylar angle		149.72 ± 14.05	149.83 ± 15.06	0.88 *
		Intercondylar distance		9.93 ± 0.61	9.92 ± 0.58	0.29 *
Class III DFD	Axial	Intercondylar angle	Mean ± SD	148.16 ± 14	144.7 ± 15.8	<0.001 *
		Intercondylar distance	Median (IQR)	10.04 (0.79)	9.95 (0.91)	0.37 ^‡^
	Coronal	Intercondylar angle	Mean ± SD	150.96 ± 13.4	150.6 ± 14.3	0.61 *
		Intercondylar distance	Median (IQR)	9.81 (0.71)	9.83 (0.7)	0.21 ^‡^

*p* values are written in bold when statistically significant; SD, standard deviation; IQR, interquartile range; * paired *t*-test; ^‡^ Wilcoxon signed-rank test; DFD, dentofacial deformity; *p* values are written in bold when statistically significant.

## Data Availability

Data are not publicly available due to data protection. Data presented in the study can be requested from the corresponding author on reasonable request and after approval of the institution.

## References

[1] Miotto A.V., Bonotto D.V., Silva J.S.C., De Souza J.F., Sebastiani A.M., Scariot R. (2023). Temporomandibular Disorders at the Preoperative Time of Orthognathic Surgery. Diagnostics.

[2] Ayyıldız E., Orhan M., Bahşi İ., Yalçin E.D. (2021). Morphometric evaluation of the temporomandibular joint on cone-beam computed tomography. Surg. Radiol. Anat..

[3] Monson L.A. (2013). Bilateral Sagittal Split Osteotomy. Semin. Plast. Surg..

[4] Holzinger D., Willinger K., Millesi G., Schicho K., Breuss E., Wagner F., Seemann R. (2019). Changes of temporomandibular joint position after surgery first orthognathic treatment concept. Sci. Rep..

[5] Bahşi I., Orhan M., Kervancıoğlu P., Yalçın E.D., Aktan A.M. (2019). Anatomical evaluation of nasopalatine canal on cone beam computed tomography images. Folia Morphol..

[6] Dalili Z., Khaki N., Kia S., Salamat F. (2012). Assessing joint space and condylar position in the people with normal function of temporomandibular joint with cone-beam computed tomography. Dent. Res. J..

[7] Krishnamoorthy B., Mamatha N., Kumar V.A. (2013). TMJ imaging by CBCT: Current scenario. Ann. Maxillofac. Surg..

[8] Zhang Z.-L., Cheng J.-G., Li G., Zhang J.-Z., Zhang Z.-Y., Ma X.-C. (2012). Measurement accuracy of temporomandibular joint space in Promax 3-dimensional cone-beam computerized tomography images. Oral Surg. Oral Med. Oral Pathol. Oral Radiol..

[9] Chenin D.L. (2010). 3D cephalometrics: The new norm. Alpha Omegan.

[10] Wen J., Liu S., Ye X., Xie X., Li J., Li H., Mei L. (2017). Comparative study of cephalometric measurements using 3 imaging modalities. J. Am. Dent. Assoc..

[11] Goncalves J.R., Wolford L.M., Cassano D.S., da Porciuncula G., Paniagua B., Cevidanes L.H. (2013). Temporomandibular Joint Condylar Changes Following Maxillomandibular Advancement and Articular Disc Repositioning. J. Oral Maxillofac. Surg..

[12] Harris M.D., Van Sickels J.R., Alder M. (1999). Factors influencing condylar position after the bilateral sagittal split osteotomy fixed with bicortical screws. J. Oral Maxillofac. Surg..

[13] Wang L.C., Lee Y.H., Tsai C.Y., Wu T.J., Teng Y.Y., Lai J.P., Lin S.S., Chang Y.J. (2021). Postsurgical Stability of Temporomandibular Joint of Skeletal Class III Patients Treated with 2-Jaw Orthognathic Surgery via Computer-Aided Three-Dimensional Simulation and Navigation in Orthognathic Surgery (CASNOS). BioMed Res. Int..

[14] Draenert F.G., Erbe C., Zenglein V., Kämmerer P.W., Wriedt S., Al Nawas B. (2010). 3D Analysis of Condylar Position after Sagittal Split Osteotomy of the Mandible in Mono- and Bimaxillary Orthognathic Surgery—A Methodology Study in 18 Patients. J. Orofac. Orthop. Der Kieferorthopadie.

[15] Chen S., Lei J., Wang X., Fu K.-Y., Farzad P., Yi B. (2013). Short- and Long-Term Changes of Condylar Position after Bilateral Sagittal Split Ramus Osteotomy for Mandibular Advancement in Combination with Le Fort I Osteotomy Evaluated by Cone-Beam Computed Tomography. J. Oral Maxillofac. Surg..

[16] Pullinger A., Hollender L. (1986). Variation in condyle-fossa relationships according to different methods of evaluation in tomograms. Oral Surg. Oral Med. Oral Pathol..

[17] Pachnicz D., Ramos A. (2021). Mandibular condyle displacements after orthognathic surgery-an overview of quantitative studies. Quant. Imaging Med. Surg..

[18] An S.-B., Park S.-B., Kim Y.-I., Son W.-S. (2014). Effect of post-orthognathic surgery condylar axis changes on condylar morphology as determined by 3-dimensional surface reconstruction. Angle Orthod..

[19] Han J.J., Hwang S.J. (2015). Three-dimensional analysis of postoperative returning movement of perioperative condylar displacement after bilateral sagittal split ramus osteotomy for mandibular setback with different fixation methods. J. Cranio-Maxillofacial Surg..

[20] Ma R.-H., Li G., Yin S., Sun Y., Li Z.-L., Ma X.-C. (2020). Quantitative assessment of condyle positional changes before and after orthognathic surgery based on fused 3D images from cone beam computed tomography. Clin. Oral Investig..

[21] Park S.-B., Yang Y.-M., Kim Y.-I., Cho B.-H., Jung Y.-H., Hwang D.-S. (2012). Effect of Bimaxillary Surgery on Adaptive Condylar Head Remodeling: Metric Analysis and Image Interpretation Using Cone-Beam Computed Tomography Volume Superimposition. J. Oral Maxillofac. Surg..

[22] Kim Y.-I., Cho B.-H., Jung Y.-H., Son W.-S., Park S.-B. (2011). Cone-beam computerized tomography evaluation of condylar changes and stability following two-jaw surgery: Le Fort I osteotomy and mandibular setback surgery with rigid fixation. Oral Surg. Oral Med. Oral Pathol. Oral Radiol. Endodontol..

